# Factors associated with past year physical and sexual intimate partner violence against women in Zimbabwe: results from a national cluster-based cross-sectional survey

**DOI:** 10.1080/16549716.2019.1625594

**Published:** 2019-06-24

**Authors:** Simukai Shamu, Patience Shamu, Mercilene Machisa

**Affiliations:** a Health Systems Strengthening Division, Foundation for Professional Development, Pretoria, South Africa; b School of Public Health, University of the Witwatersrand, Johannesburg, South Africa; c School of Clinical Medicine, Faculty of Health Sciences, Wits Reproductive Health and HIV Institute, Johannesburg, South Africa; d Gender and Health Division, South African Medical Research Council, Pretoria, South Africa

**Keywords:** Gender and Health Inequality, Intimate partner violence, partner controlling behaviour, wife beating attitudes, gender norms, women empowerment, STI and HIV

## Abstract

**Background**: Intimate partner violence (IPV) against women continues to be a public health burden globally.

**Objectives**: To assess prevalence and factors associated with women’s experiences of past 12 months physical/sexual IPV

**Methods**: A two-stage cluster-based national cross-sectional survey in which women were randomly selected for participation was conducted among 5295 women aged 15–49 years. IPV in the last 12 months was assessed using the WHO interviewer-administered questionnaire for measuring violence against women. Participants’ wife beating attitudes, partner controlling behaviours, household decision-making, STI history, HIV status and demographic characteristics were assessed. Multivariate logistic regression was conducted to assess factors associated with IPV.

**Results**: Of the 5292 women interviewed, mean age was 31.5 years and 84.7% were married. Over one-fifth of the women (20.2: 95%CI 19.1–21.3) were physically/sexually abused in the last 12 months. IPV was associated with gender inequitable norms and practices which include lacking household decision-making power (aOR 2.05, 1.71–2.47), experiencing low (aOR 2.05; 1.71–2.47) or high (aOR 4.5; 3.62–5.60) partner controlling behaviours (vs none) and endorsing low (aOR 1.29) or high (aOR 1.36) wife beating attitudes (vs none), having sexual self-efficacy (aOR 1.19; 1.10–1.41), experiencing emotional abuse (aOR 4.50; 3.62–5.60) and having a sexually transmitted infection (STI) (aOR 1.36, 1.04–1.77). IPV was also associated with women’s empowerment factors including possessing household assets (aOR 1.26, 1.03–1.54) and reporting current media usage (aOR 1.29; 1.04–1.61). Demographic factors associated with IPV were age and number of children.

**Conclusions**: This study provides evidence that IPV is a significant public health and societal problem as one in five women were abused in the past year. Younger women, less empowered women, women in inequitable intimate relationships and women endorsing traditional gender norms were at increased risk of abuse. IPV prevention programmes must prioritise transforming traditional gender norms and women’s economic empowerment.

## Background

Intimate partner violence (IPV) against women continues to be a public health burden with grave consequences globally and particularly in resource limited and gender unequal settings. A number of studies have consistently recorded high rates of IPV perpetration or experiences in Zimbabwe over the last decade with almost one in two men (44%) reported perpetrating physical and or sexual IPV [1], nearly one in two reported experiencing violence (43%) [2] while more women (46%) reported IPV during pregnancy [1–4]. Such high rates require prevention interventions to address and monitor the problem which has however been inadequately done over the last decade.

Women’s empowerment theories have been put forward to explain why some women experience IPV while others are protected from it. Some studies found that increasing women’s financial independence and access to and control of financial resources protects against violence [5]. Access to cash [6], regular employment [7], control of cash have all been associated with reduced rates of IPV [8]. The protection offered is not permanent however, as they are influenced by some other variables or contexts. For example, financial independence protects women from abuse but may pose a threat to women as it interferes with a man’s domination of a woman’s life.

Gender norms are another important risk factor for IPV. Gender norms are described as society’s social and behavioural expectations of men and women concerning their appropriate roles, rights and responsibilities [9]. Through gender theory lens, IPV is viewed as resulting from the way women are socialised to accept to be sexually passive, reliant on men for protection and economic survival and even to be disciplined by their partners when they fail to behave according to their roles [9]. On the other hand, gender norms may perpetuate the expression of toxic hyper masculinities and male dominance over female partners. Pathways between inequitable gender norms and the use of violence against female partners include how men are socialised. This leads men and women to accept and justify abuse. Many studies have found that attitudes justifying wife beating are a significant a predictor of IPV [10–12].

Assessing past IPV helps to understand recent or current IPV [13] compared to IPV that happened anytime in one’s life even if the violence has already stopped. Identifying factors associated with IPV and common characteristics among abused women or their abusers is useful in designing programmes and interventions to stop or reduce IPV [13]. It is important to do this in each setting or country as factors associated with IPV in one country may be protective in another since contexts and backgrounds differ. Although previous assessments of factors associated with IPV using DHS data have been done in a number of countries [14], factors do change with time and as such their constant update per setting is required to provide relevant up-to-date IPV information for effective IPV prevention programming. Also, the validity of measurement tools does change with time prompting changes in redefining factors and characteristics associated with IPV. Many nationally representative dedicated studies have examined risk factors for women’s experiences of IPV globally [13,15–20]. Fewer national and dedicated studies have been conducted in African settings [21]. This makes it difficult to understand the drivers of IPV at a national level as well as to compare these between countries. However, the DHS programme has periodically implemented the Domestic Violence Module in some countries including Zimbabwe and is generating waves of data that is useful to understand trends and the influences of different factors. In this paper, we assessed the prevalence and factors associated with women’s experiences of past-year physical/sexual IPV using data collected in the 2015 Zimbabwe Demographic Health Survey (ZDHS 2015).

## Methods

### Study design

The data for this study came from the Zimbabwe Demographic and Health Survey (ZDHS) conducted in 2015 in all the 10 provinces of Zimbabwe [22]. This was a national cross-sectional survey of women and men aged 15 years and above. We extracted data for women aged 15–49 years who participated in the domestic violence module of the ZDHS.

### Sampling

A two-stage stratified sampling design was employed by rural/urban status and province. Each household had an equal probability of selection. The first stage was a census enumeration area (subdivision of administrative ward in a district in a province) of which there were 400 enumeration areas (166 in urban and 234 in rural areas) in total. The second stage was the household where households were picked randomly from all listed private dwellings in each enumeration area. One woman per household was randomly selected for an interview on IPV. All women were offered an opportunity to draw a blood specimen for laboratory HIV testing.

### Data collection

Trained interviewers used tablets to record responses during interviews and uploaded data electronically through Bluetooth technology on census and surveys processing (CSPro) managed by the DHS programme in collaboration with the US Census Bureau. A pre-test was conducted after training interviewers. Data were collected between July and December 2015. Of a total of 11,196 households, 10,657 were occupied and 10,534 were interviewed giving a response rate of 98.8%. Eligible women response rate was 96.2% (9955/10351). Of these, 7223 women were interviewed for domestic violence and 5494 had ever had a partner and were therefore interviewed for IPV. Of these, 5295 (96.4%) responded to physical and sexual IPV questions, and our analysis is based on this sample.

### Measures

The questionnaire contained a number of measures, shown in Table 1, which include the following: **Demographic questions** included the women’s and partner’s ages, highest educational level completed, employment in the past 12 months, current marital status, number of unions ever entered and material items they possessed in their house (radio, car, TV, bicycle, telephone, motorcycle, fridge, land, house). **Intimate partner violence (IPV)** was assessed using the WHO measures of violence against women and girls [23] built on the revised version of the Conflict Tactics Scale [24] **Physical IPV** was measured by seven questions that asked a woman if a partner had perpetrated any acts of physical violence namely pushed, shook or threw something at her, slapped, punched her with a fist, kicked or dragged, strangled or burnt her, threatened her with a knife or gun in the last 12 months. **Sexual violence** was measured by three questions that asked a woman if a partner had forced her to have sex when she did not want to, to have an unwanted sex act, or forced her to perform a sexual act that she did not want to in the last 12 months. Women who confirmed experiencing any of the acts of sexual violence were considered to have experienced sexual IPV in the last 12 months. Physical and or sexual abuse was assessed as having experienced any of the defined acts of physical or sexual IPV in the last 12 months. **Emotional violence** was measured by three questions that asked a woman if a partner ever humiliated, threatened, insulted, or made her feel bad in the last 12 months. A binary variable was created for women who experienced any of the listed acts of emotional violence vs those who experienced none of these violent acts.

**Table 1.** showing the key measures used in the study.

	Measures	No of items	Typical item	Source
1	Physical intimate partner violence	7	Did your (last) (husband/partner) ever kick you, drag you, or beat you up? Responses were Yes or No. If yes they were asked: How often did this happen during the last 12 months: often, only sometimes, or not at all?	Revised CTS, WHO, DHS
2	Sexual Intimate partner violence	3	Did your (last) (husband/partner) ever physically force you to have sexual intercourse with him when you did not want to? Responses were Yes or No. If yes they were asked: How often did this happen during the last 12 months: often, only sometimes, or not at all?	Revised CTS, WHO; DHS
3	Emotional Intimate partner violence	3	Did your (last) (husband/partner) ever threaten to hurt or harm you or someone you care about? Responses were Yes or No. If yes they were asked: How often did this happen during the last 12 months: often, only sometimes, or not at all?	Revised CTS, WHO; DHS
4	Partner controlling behaviours	5	He (is/was) jealous or angry if you (talk/talked) to other men? Responses were 1 = Yes, 0 = No	WHO, DHS
5	Wife beating attitudes	5	Is a wife justified in refusing to have sex with her husband when she knows he has sex with other women? Responses were 1 = Yes, 2 = No	Saunders, Lynch, Grayson, Linz 1987 Violence and Victims; DHS
6	Decision- making	4	Who usually makes decisions about health care for yourself: you, your husband/partner, you and your husband/partner jointly, or someone else? Responses were 1 = Respondent, 2 = Husband/partner, 3 = Respondent and husband/partner jointly, 4 = someone else, 5 = Other	DHS
7	Media frequency use	3	Do you listen to the radio at least once a week, less than once a week or not at all? Responses were: 1 = at least once a week, 2 = less than once a week, 3 = not at all	DHS
8	Assets possession	8	Do you have a television? Responses were: 1 = Yes, 0 = No	DHS
9	HIV	1	*A first assay test (ELISA, the Vironostika® HIV Ag/Ab (fourth generation) (Biomerieux)) was confirmed with a second ELISA, (the Enzygnost® HIV Integral II, fourth generation) while the Western Blot (DiaSorin), was used to confirm discordant results	See *
10	Sexual Self-efficacy	1	Can you say no to your (husband/partner) if you do not want to have sexual intercourse? Responses were 1 = Yes, 2 = No, 3 = depends/not sure	DHS
11	STI	3	Sometimes women experience a bad-smelling abnormal genital discharge. During the last 12 months, have you had a bad-smelling abnormal genital discharge? Responses were 1 = Yes, 0 = No	WHO, DHS
12	Multiple sexual partners	1	In total, with how many different people have you had sexual intercourse in the last 12 months?	WHO, DHS

A woman’s **decision-making power** [25] (Cronbach’s alpha = 60.4%) was measured by indicators of intra-household decision-making and as a proxy for her empowerment or bargaining power. This was assessed by asking who has a say on key issues in the household, on her own health care, large household purchases, visits to relatives/family and deciding what to do with the money the husband/partner earns. Responses were respondent alone = 1, both respondent and partner = 2, husband/partner alone = 3, someone else = 4 and other = 5. We combined the four questions and added the responses. A lower average response indicated high decision-making power while a higher mean score indicated lower decision-making power in the household. We used a mean score of 4 (in all four domains) to indicate high power, 5–8 medium and 9+ low decision-making power. **Sexual self-efficacy**: Women were asked if they could refuse sex for any reason as shown in Table 1. Those who responded affirmatively (Yes) were regarded as having sexual self-efficacy. We measured **partner controlling behaviour** [23] (Cronbach’s alpha = 55.4%) using five questions that asked if a husband is jealous, if her partner talks to other men, husband accuses her of unfaithfulness, does not permit her to meet her female friends, tries to limit her contact with family or does not trust her with money. We constructed a variable that had no control = 0 control issues, 1 = 1–2 control measures (low level of control) and 3 = at least three control measures showing a high level of partner control. We assessed women’s **attitudes towards wife beating** [26] (Cronbach’s alpha = 76.3%) by asking women through five questions if a man is justified for beating his wife if she goes out without telling him, neglects the children, argues with him, refuses to have sex with him or burns the food. Responses were 1 = Yes or 0 = No. By summing the responses we created an attitudes scale. A higher score was indicative of more accepting attitudes towards wife beating. We also created a binary variable for the gender attitudes scale with a score of ≤2 being low acceptance and a score of 3+ being considered as higher acceptance. **Media usage**: We asked women how often they watched the television, listened to the radio and read newspapers and magazines. Responses were summed up to have a media usage scale. We added the scores across the three variables 1 = not at all; 2 = less than once a week (low media use) and 3 = at least once a week (high media use). **Sexual factors**: We assessed if a woman ever had an **STI** in the last 12 months by asking three questions – if she ever had a genital discharge, genital sore or an STI in the last 12 months. A Yes response to any of these questions was regarded as ever had an STI in the last 12 months. HIV status was determined by collecting a blood sample from each participant and testing it in a laboratory using ELISA. The samples were checked for accuracy using a second ELISA test and in the case of discordant results, Western Blot was used to resolve the conflict in a third test – see Table 1. Participants were asked about the number of **sexual partners** they had in the last 12months and age at which they first had sexual intercourse.

### Data analysis

The DHS data specially adjusted for the selection of only one woman per household selected for interviews throughout the country. In addition, specially constructed weights were used in adjusting for this selection. This ensured that the selected individuals were nationally representative. All data analysis was conducted in Stata 13.0. We calculated frequencies of IPV and presented them in percentages and 95% confidence intervals. We used chi-square analysis to assess the independent relationships between demographic variables and physical and/or sexual IPV. This was also done on behavioural and sexual risk-related variables – STI, sex partners, HIV variables. We categorised severe physical violence as being kicked or dragged, strangled or burned, threatened with or partner actually used a weapon and less severe physical violence as being pushed, shook or threw something at her, slapped, punched, twisted arm or pulled hair. We conducted a multivariate logistic regression analyses to assess factors associated with experience of physical and or sexual IPV in the last 12 months. Multivariable models included variables associated with IPV from the literature [26–28] as well as those showing statistically significant associations in bivariate analysis. Variables that showed a p > 0.250 in bivariate analysis were excluded from multivariable models. We checked for collinearity and the mean (3.74) variance inflation factor showed there was no collinearity deserving investigation. Models also controlled for a woman’s demographic characteristics and ever experience of violence. Results are presented as adjusted odds ratios (aORs) and CIs (See Table 5.

**Table 2.** Prevalence of past year IPV experience (*N* = 5292).

	*n*/*N*	%	95%CI
Emotional violence	1263/5292	23.9	22.7–25.0
Sexual violence	471/5292	8.9	8.1–9.7
Physical violence	842/5292	15.9	14.9–16.9
Physical and/or sexual violence	1068/5292	20.2	19.1–21.3
Severe physical violence	329/5292	6.2	5.6–6.9
Less severe physical violence	812/5292	15.3	14.4–16.3
Physical, sexual and/or emotional violence	1495/5292	28.3	27.0–29.5

**Table 3.** Socio-demographic characteristics of the sample by experience of past year physical and/sexual IPV (*N* = 5292).

	Total		Never physical and/sexual IPV		Physical and/sexual IPV once or more		
	*n*	%	*n*	%	*n*	%	*p* Value
**Age:**							
15–24 years	1200	22.7	754	21.91	446	24.1	
25–34 years	2251	42.54	1412	41.03	839	45.3	
35+ years	1841	34.79	1278	37.05	566	30.58	<0.0001
**Education:**							
Up to primary level	1577	29.8	985	28.6	592	31.9	0.011
At least secondary level	3715	70.2	2456	71.37	1259	68.02	
**Total children ever born** (ref = 0)	283	5.35	212	6.16	71	3.84	
1–2 children	2273	43.0	1475	42.87	798	43.11	
3+ children	2736	51.7	1754	50.97	982	53.05	0.001
**Marital status**: Married and/or living with a partner	4484	84.73	2979	86.57	1505	81.31	<0.0001
**No of unions in lifetime**: more than once	568	16.51	342	18.48	910	17.2	0.070
**Partner’s age:**							
15–34 years	1945	43.4	1233	41.42	712	47.3	
35+ years	2537	56.6	1744	58.58	793	52.69	<0.0001
**Partner’s education**: At least secondary level	3506	78.2	2350	78.9	1156	76.8	0.103
**Partner not employed last 12 months**	635	14.17	437	14.68	198	13.16	0.044
**Living in urban area (vs rural)**	2132	40.29	1384	40.22	748	40.4	0.893
**Possession of assets** (radio, car, TV, bicycle, telephone, motorcycle, fridge)							
0 items	1412	27.5	923	27.63	489	27.26	
1–2 items	2425	47.2	1537	46	888	49.5	
3–8 items	1298	25.28	881	26.37	417	23.24	0.023

**Table 4.** Characteristics of the sample by physical and/sexual abuse (*N* = 5292).

	Total		Never IPV		IPV Once or more		
	*n*	%	*n*	%	*n*	%	
**Decision- making:**							
Woman alone	178	4.03	130	4.43	48	3.24	
Jointly with partner	3027	68.55	2087	70.08	940	63.51	
Does not make decisions	1211	27.42	719	24.49	492	33.24	<0.0001
**Media usage (print, radio, television):**							
Never	1258	23.77	847	24.61	411	22.2	
Low frequency	1455	27.49	943	27.4	512	27.66	
High frequency	2579	48.73	1651	47.98	928	50.12	0.128
**Justifies wife beating for any reason:**							
No	3383	64.87	2326	68.53	1057	58.05	
1–2 items only	1218	23.36	726	21.39	492	27.02	
3–5 items	614	11.77	342	10.08	272	14.94	<0.0001
**Sexual Self-efficacy (ref = No)**	3238	72.24	2115	71.04	1123	74.62	0.031
**Partner’s controlling behaviours:**							
No	1756	33.18	1481	43.04	275	14.86	
Low level of control	2249	42.5	1513	43.97	736	39.76	
High level of control	1287	24.32	447	12.99	840	45.38	<0.0001
**Emotional violence in the last 12 mo (ref = No)**	1659	31.35	525	15.26	1134	61.26	<0.0001
**Age at first sex:**							
Below 15 years	329	6.27	184	5.4	145	7.88	
15+ years	4920	93.73	3225	94.6	1695	92.12	<0.0001
Multiple sexual partners in the last 12 months	4067	77.13	2684	78.34	1383	74.88	0.004
**Had an STI in the last 12 months (ref = No)**	447	8.45	226	6.57	221	11.94	<0.0001
**HIV positive status (ref = Negative)**	1135	21.45	718	20.87	417	22.81	0.145

**Table 5.** Multivariate logistic regression showing factors associated with intimate partner violence (*N* = 5292).

Variable	aOR	95% CI	
**Age: 35+ years (ref = 15–24 years)**			
Age: 25–34 years	0.77	0.60–0.98	
Age: 35+ years	0.57	0.40–0.80	
**Partner’s age 35+ years (ref = 15**–**34years)**	0.67	0.53–0.84	
**Total children ever born ref = 0**			
1–2 children	1.57	1.09–2.26	
3+ children	2.13	1.45–3.14	
**Owns a house/land (ref = jointly owns with partner)**	1.24	1.05–1.46	
**Possession of assets ref: no item**			
1–2 items only	1.26	1.03–1.54	
At least 3 items	0.98	0.76–1.26	
**Decision-making: ref = Usually makes decisions alone**			
Usually makes decisions jointly with partner	1.26	0.84–1.89	
Does not make decisions	2.05	1.71–2.47	
**Media usage (print, radio, television) ref = not at all**			
Low frequency	1.11	0.89–1.39	
High frequency	1.29	1.04–1.61	
**Had an STI in the last 12 months (ref = No)**	1.36	1.04–1.77	
**Justifies wife beating for any reason (ref = 0 instances)**			
1–2 items only	1.36	1.13–1.62	
3–5 items	1.62	1.28–2.054	
**Woman can refuse sex (ref = No)**	1.19	1.01–1.41	
**Emotional violence in the last 12mo (ref = No)**	5.06	4.31–5.96	
**HIV Positive status (ref = Negative)**	1.08	0.88–1.31	
**Controlling behaviours (ref = No)**			
Low level of control	2.05	1.71–2.47	
High level of control	4.50	3.62–5.60	

## Results

A total of 5292 women had partners and were interviewed for IPV. Mean age was 31.5 years (95%CI: 31.3–31.7), 70.2% completed at least secondary education (11 years of formal education), 84.7% were married and or lived with partners, and 94.7% had at least one child and their partners mean age was 37.9 years (95% CI: 37.5–38.2) and most (78.2%) were educated to at least secondary level.

Table 2 shows the prevalence of different types of IPV in the last 12 months. More than one in five (20.2%) of the women reported physical and or sexual IPV. 15.9% reported physical abuse and 8.9% reported sexual abuse. A participant could report either, or both types of IPV and this counted as having been physically and/or sexually abused. Almost a quarter of the women (23.9%) reported experiencing emotional abuse. 28.3% of the women reported any physical, sexual and/or emotional IPV. Severe physical violence in the last 12 months was reported by less than a tenth of women (6.2%) while 15.3% reported less severe physical abuse in the last 12 months.

Table 3 shows socio-demographic characteristics of the sample by experience of past year physical and/sexual abuse. Younger women reported a higher prevalence of abuse than the older (35+ years) women (p < 0.0001). Less educated women who only had a primary education reported a higher prevalence of IPV experiences than women who had higher than primary education (p = 0.011). Experiences of IPV were higher among women who were married or cohabiting with their partners compared to women who were not living with their partners (p < 0.0001). Women who had younger partners aged 15–34 years reported a higher prevalence of abuse compared to those with older partners of ages 35+ years (p < 0.0001). Experiences of IPV were lower among women whose partners were unemployed compared to women whose partners were employed in the last year (p = 0.044). A higher proportion of women who possessed fewer household items (1–2) were abused (p = 0.023). Participants’ exposure to violence did not differ by rural or urban divide (p = 0.893).

Table 4 shows behavioural and sexual risk characteristics of the sample by experience of past year physical and/sexual abuse. A higher proportion of the women who never made any household decisions reported abuse than those who made decisions (p < 0.0001). More abused women had more accepting wife beating attitudes (p = 0.0001) and higher sexual self-efficacy than those who did not report abuse (0.031). More abused women reported high levels of partner controlling behaviours than the non-abused women (p < 0.0001). More abused women reported sexual debut before age 15 than the non-abused women (7.9% vs5.4%, p < 0.0001). A smaller proportion of the abused women reported multiple sexual partners than those who did not report abuse (p = 0.004). More abused women reported testing positive to STIs in the last 12 months (11.9%vs 6.6%p < 0.0001) and positive to HIV in the study (22.8% vs20.8%; p = 0.145) than those who were not abused.

Table 5 shows factors associated with physical and/or sexual abuse in the last 12 months. Results show that when compared to younger women aged 15–24, older women (25 years and above) had lower odds of being abused (aOR 0.77 95% CI: 0.60–0.98 for 25–34 years old and aOR 0.57 95% CI: 04.40–0.80 for those 35+ years). Women with partners aged 35 years or older were also protected from abuse compared to those with younger partners (aOR 0.67, 95%CI 0.53–0.84). Compared to women with no children, those with up to two children had higher odds of being abused (aOR 1.57; 95%CI 1.09–2.26) while having at least three children had the highest odds of reporting abuse (aOR 2.13 95%CI 1.45–3.14).

We assessed the association between IPV and empowerment factors which include household possession of assets and media use. Reporting possession of one to two household assets was associated with experiencing abuse compared to not possessing any of the eight listed household items (aOR 1.26, 95%CI 1.03–1.54). Having three or more possessions was protective of violence although it did not reach statistical significance (aOR 0.98). Women with a high frequency of media use had higher odds or reporting abuse than those with no media exposure (aOR 1.29; 1.04–1.61).

A strong relationship between decision-making in the household and experiencing violence was demonstrated in the logistic regression analysis. Women who reported not making any decisions had higher odds of reporting abuse compared to women who usually made decisions alone (aOR 2.05, 95%CI 1.71–2.47) while those who jointly made decisions with their partners had higher odds of reporting abuse although it did not reach statistical significance (aOR1.26; 0.84–1.89).

The association between IPV and gender roles and attitudes was assessed using IPV as the independent variable and decision-making, wife beating attitudes, sex refusal, partner controlling behaviours experiencing emotional violence as well as ever having an STI/HIV as the dependent variables. We found an association between justifying wife beating and IPV experience. When compared to women who did not justify abuse, those who justified abuse in 1–2 instances had higher odds of reporting abuse (aOR 1.29). A dose–response relationship was found as the highest odds were found on women who justified abuse in at least three instances. (aOR 1.36). Reporting sexual self-efficacy was positively associated with reporting abuse (aOR1.19). A higher level of partner controlling behaviours was positively associated with abuse and this had a dose–response relationship as those reporting high levels of control had higher odds of reporting abuse (aOR 4.5; 3.62–5.60) followed by those reporting a relatively lower level of control (aOR2.05; 1.71–2.47). Having experienced emotional abuse had the strongest odds of experiencing abuse – a five-fold odds of being physically/sexually abused (aOR 5.06; 4.36–5.96). Among the sexual factors assessed a history of STI in the last 12 months was associated with experiencing IPV (aOR 1.36, 1.04–1.77) while a positive association with an HIV positive status was found although it did not reach significance level.

## Discussion

In this paper, we aimed to assess the prevalence and factors associated with women’s experiences of physical and/or sexual abuse in the last 12 months using data from the ZDHS 2015. We showed evidence that IPV is a considerable public health and societal problem with more than one in five of the participating women reporting physical and/sexual IPV experiences in the past year. Independently owning resources, or just owning 1–2 of the listed household assets were associated with increased abuse. Being economically empowered through the use of the media and owning household assets was associated with experiencing IPV. Women who experienced partner controlling behaviours, those who did not have household decision-making power, justified wife beating or reported refusing sex with their partners were more likely to report IPV experiences. Regarding demographic characteristics, younger women or women in relationships with younger men as well as those with more children were more likely to report recent IPV experiences.

Indicators of empowerment were differentially associated with women’s IPV experiences. Non-involvement in household decision-making was associated with increased IPV. Yet women who independently owned a house, land or owned one or two household assets were more likely to report IPV experiences. Moreover, women with higher exposures of media reported a higher prevalence of IPV. Media has been used as an advocacy tool in the fight against gender-based violence [29] and the higher reporting of IPV by women highly exposed to media consistently correlates with increased knowledge and awareness of social issues in the community. Secondly, the media is used to positively change attitudes for healthy behaviours which in the case of gender-based violence includes disclosing experiences and seeking help for it [30]. Our findings indicate the potential power of media to challenge the social stigma and change the culture of silence that often makes women victims of IPV not disclose or seek informal or formal support. Our study also indicates that economic empowerment of women such as access to land or houses may also positively lead to higher disclosures of violence [5]. There is a notion that women who are economically dependent on abusive partners are less likely to disclose IPV out of desperation and fear of abandonment [31,32]

Our study indicated a positive association between sexual resistive efficacy and IPV. This may be explained in two ways. Firstly, women’s ability to refuse sex may be an indicator of their progressiveness and empowerment which may in turn make them more likely to disclose abuse compared to less progressive or empowered women. Secondly, it cannot be ruled out that being progressive or liberal may place women at increased odds of IPV when they assert their beliefs in their intimate relationships. There is evidence that IPV occurs because of relationship conflict which may include man asserting their dominant position over women. In the case where men feel sexually entitled, women who are liberal to refuse sex pose as challenging men’s authority and dominance in traditionally defined male domains, so rendering themselves at increased odds of IPV experiences. Previous studies have reported men’s perpetration of sexual violence in Zimbabwean against women who resist male sexual power and dominance [33]. Jewkes argued elsewhere that increasing use of empowering knowledge to challenge male dominance such as refusing sex carries with it violence as a form of correcting and resisting this behaviour until a high level of empowerment is reached to challenge male power without receiving violence in return [27]. Although this explanation is highly probable in our study, we cannot apply it with certainty since our research was a cross-sectional study. Prospective longitudinal studies with multiple years of follow up, although expensive to conduct, can ascertain this.

Emotional violence was strongly linked to physical and or sexual abuse of women in our study. Previous studies also reported a link between the two forms of violence [34]. It could be that emotional violence degenerates into physical/sexual violence or vice versa or that the two simultaneously occur in a violent relationship. Emotional violence is an important measure of IPV [35] as it informs us more about the state of the relationship between partners.

IPV is largely influenced by societal influences which include social structure and gender norms about what a true or real man or woman is and the gender norms that put women in a subordinate and passive position and contrastingly putting men in a dominant and active position. In some cultures beating a woman is regarded as normal disciplining or correcting a woman and a sign that the man loves his partner [36]. Our study’s finding that more women who justified wife abuse reported IPV illustrates such gender norms which perpetuate violence. It also shows that women are socialised into believing that a wife can be beaten for some reason and that it is normal. De-socialisation of violence as a means of instituting discipline is required and work with young people to prevent and transform attitudes is required in this regard.

Our study found that having an STI was associated with experiencing physical/sexual abuse. Similar findings were reported in a Zimbabwean study before [3] and the circumstances in which this happen have also been described qualitatively as related to poverty and male dominance [33] that become prime over protecting oneself against contracting an STI. Similar studies in Africa have found male dominance and sexual entitlement influencing such relations [37]. Although HIV status is not always related or does not always reach significance levels with violent experiences [38], the fact that having an STI, it being a strong risk factor for HIV shows that sexual factors are strongly related to intimate partner violence [39,40]. STIs which signify non-use of protection during sex illustrate women’s failure or inability to negotiate safe sex with their partners due to fear of several things including being blamed by the partner, being rejected or a clear use of male force during sex.

Despite some scholars arguing that age is not associated with IPV [27], our study found older age to be an important protective factor as older women and women with older partners were protected from IPV. This is consistent with findings from other studies which found negative associations between age and IPV in older populations [28] as well as prospective studies that found violence decreasing with age [41]. It could be that young people lack conflict management skills which they then learn with time thereby reducing the frequency of IPV as they transit into older ages. Intervention studies should, therefore, target younger people with IPV prevention and conflict management skills to prevent IPV. Work with men and boys is key to addressing gender-based violence in relationships.

The number of children is a long-standing risk factor for IPV [27]. However, careful interpretation of this risk factor is required as it is not necessarily correct that children have a direct influence on a woman’s experience of violence. Rather, the circumstances of having a child that usually repeat or increase with each child or conception trigger situations where men use violence due to failure to negotiate peaceful ways of arguing and addressing real family challenges [42]. Earlier studies in Zimbabwe found that the behavioural and economic demands to support a pregnancy or childbirth require more understanding from men who unfortunately traded requirements for pregnancy needs with violence if they could not provide the required resources [33]. The pregnancy setting triggers violence. In one study an additional pregnancy was associated with 10% increase in experiences of IPV [43]. In addition, partner unwillingness to use birth control and expressions of inability to afford contraception have often been associated with women’s increased IPV experiences [43]. In our study, a number of children had a dose–response relationship with IPV experiences. Increased number of children may have posed increased challenges brought about by pregnancy challenges or challenges of looking after a bigger family compared to a smaller family or not having a child at all [44]. Given that poverty in Zimbabwe reached unprecedented levels with unemployment projected at 80% during the time of the study, this explanation is highly probable. However, more research is required to ascertain the nature of the association.

The limitations of the DHS data have been described elsewhere [38] and include that it was a cross-sectional study which limits causality explanation and so we cannot be certain that the factors that we found were causes of the abuse we measured as the two may have happened simultaneously or that the violence may have occurred before factors considered as independent variables in the study occurred. Also, using data that were not collected for the purpose of the stated objective runs the risk of not perfectly being able to assess the objective of the analyses. This is because some of the variables that we would have wanted to analyse were not available and those available were not in the format that we would have wanted for our analysis.

The study did not explore the contribution of economic pressures to relationship conflict and IPV. Based on the observed relationship of higher IPV among women who have birthed more children, we have assumed that a harsh economic climate and high unemployment is a possible mediator. However, because of the cross-sectional design, the study is limited in establishing temporality. It may be that women who experience lifetime IPV are less likely to exercise birth control hence conceive and have more children. Future studies should explore this further. Similarly, the directionality of the associations of IPV with economic empowerment variables is not clear i.e. whether economic empowerment leads to increased reporting of IPV or leads to increased experiences of IPV. Longitudinal research may be necessary to understand these relationships better. Future studies should also employ path analyses to fully understand the relationships and mediation of variables.

The study, however, was cluster-based, participants were randomly selected to participate in the domestic violence sub-study and that it was a national study with a very high response rate and used a tested questionnaire – all these constitute the strengths of the paper. In addition, our findings are aligned with what most previous studies found. Assessing past 12 months IPV is a strong measure of recent IPV and frequency of IPV compared to studies that use lifetime measures of IPV which are general and increase challenges of association temporality.

## Conclusions

This study provides evidence that IPV is a significant public health and societal problem in Zimbabwe. One in five women from a national population-based study reported experiencing physical/sexual IPV in the past year. The study also found high rates of emotional violence. Younger women, less empowered women in terms of household possession of assets or household decision-making, women in inequitable heterosexual intimate relationships and women endorsing traditional gender norms were at increased risk of IPV experiences. Gender violence prevention programmes must prioritise approaches that include sexuality education and emancipation, transforming traditional gender norms and economic empowerment of women. Work with men and boys to promote gender equitable relationships continues to be a critical and necessary IPV prevention approach.

## Paper context

What is already known: Intimate partner violence is a public health burden with serious health and social problems. **What this paper adds**: More than one in five women are abused. Less empowered women, women in inequitable intimate relationships and women endorsing traditional gender norms were at increased risk of past year violence **What action needs to be taken**: Transforming traditional gender norms, sexuality education and women’s economic empowerment are urgently needed to prevent partner violence.
